# The gut microbiome and resistome of conventionally vs. pasture-raised pigs

**DOI:** 10.1099/mgen.0.001061

**Published:** 2023-07-13

**Authors:** Devin B. Holman, Katherine E. Gzyl, Arun Kommadath

**Affiliations:** ^1^​ Lacombe Research and Development Centre, Agriculture and Agri-Food Canada, 6000 C&E Trail, Lacombe, AB, T4L1W1, Canada

**Keywords:** antimicrobial resistance, metagenomics, pasture-raised, pigs

## Abstract

Conventional swine production typically houses pigs indoors and in large groups, whereas pasture-raised pigs are reared outdoors at lower stocking densities. Antimicrobial use also differs, with conventionally raised pigs often being exposed to antimicrobials directly or indirectly to control and prevent infectious disease. However, antimicrobial use can be associated with the development and persistence of antimicrobial resistance. In this study, we used shotgun metagenomic sequencing to compare the gut microbiomes and resistomes of pigs raised indoors on a conventional farm with those raised outdoors on pasture. The microbial compositions as well as the resistomes of both groups of pigs were significantly different from each other. Bacterial species such as *Intestinibaculum porci *, *Pseudoscardovia radai* and *Sharpea azabuensis* were relatively more abundant in the gut microbiomes of pasture-raised pigs and *Hallella faecis* and *Limosilactobacillus reuteri* in the conventionally raised swine. The abundance of antimicrobial resistance genes (ARGs) was significantly higher in the conventionally raised pigs for nearly all antimicrobial classes, including aminoglycosides, beta-lactams, macrolides-lincosamides-streptogramin B, and tetracyclines. Functionally, the gut microbiomes of the two group of pigs also differed significantly based on their carbohydrate-active enzyme (CAZyme) profiles, with certain CAZyme families associated with host mucin degradation enriched in the conventional pig microbiomes. We also recovered 1043 dereplicated strain-level metagenome-assembled genomes (≥90 % completeness and <5 % contamination) to provide taxonomic context for specific ARGs and metabolic functions. Overall, the study provides insights into the differences between the gut microbiomes and resistomes of pigs raised under two very different production systems.

## Data Summary

All metagenomic sequences and metagenome-assembled genomes are publicly available in the National Center for Biotechnology Information’s (NCBI) sequence read archive and genome databases under BioProject PRJNA857725.

**Impact Statement**

Antimicrobial use in swine production continues to be a serious concern due to its potential association with the development and persistence of antimicrobial resistance. Most commercial pigs in North America are raised indoors at high stocking densities whereas a smaller segment of the swine sector raises pigs entirely outdoors on pasture. However, very little is known about how these two management systems affect the pig gut microbial community and antimicrobial resistance. In this study we show that pigs raised outdoors on pasture have very different gut microbiomes compared to conventionally raised pigs housed indoors. Furthermore, even in the absence of direct antimicrobial exposure, the conventionally raised pigs carried a greater abundance of antimicrobial resistance genes in their gut.

## Introduction

Pigs raised on conventional swine farms are typically housed indoors in large numbers under tightly controlled environmental conditions. Conversely, pasture-raised pigs are usually kept outdoors in smaller numbers where they have access to the soil and plants growing in the pasture. Antimicrobial use also often differs between these two production systems. Unlike conventionally raised pigs, pasture-raised pigs are much more likely to be treated individually if required rather than as a group if an animal becomes ill. Antimicrobial use in food-producing animals such as swine has come under increased scrutiny due to its potential association with antimicrobial resistance [1]. However, even in the absence of antimicrobial exposure conventionally raised or antimicrobial-free pigs raised indoors frequently still have a relatively diverse and rich resistome [2–4]. The resistome refers to antimicrobial resistance genes (ARGs) in all bacteria within a given environment and includes both acquired and intrinsic ARGs [5].

The gut microbiome plays an important role in the health and performance of swine. For example, the gut microbiome encodes many genes involved in the metabolism of non-digestible dietary carbohydrates into metabolites such as short-chain fatty acids (SCFAs) that can be used as an energy source by the host [6]. The swine gut microbiome is largely shaped by diet but is also influenced by age, antimicrobial use, disease state, environment and genetics [4, 7, 8]. Cultivation of many microorganisms found in the mammalian gut remains challenging as their growth requirements are often unknown; however, techniques such as shotgun metagenomic sequencing can provide new insights into both the function and composition of microbiomes without the need for extensive culturing.

Pigs raised outdoors on pasture and without prior exposure to antimicrobials can provide important insights into how management practices can influence the swine gut microbiome and resistome. Therefore, our objective here was to characterize the gut microbiomes and resistomes of commercial pigs that had been raised entirely outdoors without antimicrobial exposure and to compare them with conventionally raised pigs that also did not receive antimicrobials.

## Methods

### Animals and sampling

The pasture-raised pigs were crossbred Duroc, Tamworth, Berkshire and Large Black breeds located on a commercial operation in Alberta, Canada, where they were housed outdoors year-round and did not receive antimicrobials. Faecal samples were collected from pigs in various production stages: sows (*n*=13), nursery (*n*=10) and growing/finishing (*n*=6). Faecal samples were also taken from Duroc × Landrace × Yorkshire nursery (28 days of age; *n*=6) and growing/finishing (84 and 140 days of age; *n*=11) pigs that were weaned at 21 days of age as well as sows (*n*=10) at the swine unit of the Lacombe Research and Development Centre. These pigs were housed together in pens with six animals per pen and did not receive antimicrobials.

### Metagenomic DNA extraction and sequencing

Metagenomic DNA was extracted from 130 mg of faeces using the QIAamp BiOstic bacteremia DNA kit (Qiagen) as previously described [4]. DNA was quantified using the Quant-iT PicoGreen dsDNA Assay Kit (Thermo Fisher Scientific) and metagenomic libraries were prepared using 50 ng of DNA and the NEBNext Ultra II DNA Library Prep Kit (New England BioLabs) as per the manufacturer’s recommendations. Size selection and purification of the metagenomic libraries was carried out using SparQ PureMag beads (Quantabio) and an Illumina Genome Analyzer (Illumina) with a KABA SYBR Fast Universal qPCR Kit (Kapa Biosystems) was used for quantification. The metagenomic libraries were normalized and pooled and 225 pM was sequenced together with PhiX (1 %) on a NovaSeq 6000 with a S4 flow cell (300 cycles) as per the manufacturer’s instructions. DNA from a mock community of 20 bacterial strains (MSA-2002; ATTC) was also extracted and sequenced as a positive control.

### Metagenomic sequencing analysis

Low-quality reads and sequencing adapters were removed with fastp v.0.23.2 [9] using a 4 bp sliding window and a quality threshold of 15. Any reads of less than 100 bp were removed. Swine host and PhiX sequences were removed using Bowtie2 v.2.4.5 [10] to align the reads to three swine genome assemblies [Sscrofa11.1 (Duroc]), Berkshire_v1 (Berkshire), USMARCv1.0 (Duroc × Landrace × Yorkshire)] and the *Escherichia* phage phiX174 genome (NC_001422) for removal. SAMtools v.1.14 [11] and BEDtools v.2.30.0 [12] were used to extract reads that did not align to the swine and PhiX genomes and these paired fastq reads were used for all subsequent steps. Taxonomic classification of the metagenomic samples was carried out using Kaiju v.1.9.0 [13] and the NCBI non-redundant protein database (19 March 2022). The unassembled reads were also screened for ARGs using the resistance gene identifier (RGI) v.6.0.1 and the Comprehensive Antibiotic Resistance Database (CARD) v.3.2.5 [14] with KMA [15] used for alignment. Carbohydrate-active enzyme (CAZyme) profiles were characterized by mapping the reads to the dbCAN2 [16] v.09242021 using DIAMOND v.2.0.14 [17] (≥90 % amino acid identity and ≥90 % coverage). Copper and zinc resistance genes were identified using DIAMOND (≥90 % amino acid identity and ≥90 % coverage) and the BacMet antibacterial biocide and metal resistance genes database v.2.0 [18].

### Metagenome-assembled genomes

Metagenomic sequences from conventionally and pasture-raised pigs were co-assembled separately as well as individually assembled using MEGAHIT v.1.2.9 [19]. Each sample was mapped back to its respective assembly and the co-assembly using Bowtie and the contigs (≥2000 bp) were binned into metagenome-assembled genomes (MAGs) using MetaBat 2 v.2.2.15. The completeness and contamination of both the co-assembled and individually assembled MAGs were calculated with CheckM v.1.1.10 [20]. A total of 19 482 MAGs were recovered with >90 % completeness and <5 % contamination. These MAGs were then dereplicated using dRep v.3.2.2 with primary clustering set at 90 % and secondary clustering set at 99 %, resulting in 1043 MAGs for subsequent analyses. Taxonomy was then assigned to these 1043 dereplicated MAGs using GTDB-Tk v.2.1.1 [21] and the GTDB database release 207 [22].

Genes in the MAGs were predicted and annotated using Distilled and Refined Annotation of Metabolism (DRAM) v.1.3.5 [23] with the dbCAN2 HMMdb release 10.0 and KEGG release 101.0 databases for annotation of CAZymes and other metabolic pathways, respectively. The MAGs were also screened for ARGs using the RGI with the CARD and for copper and zinc resistance genes using DIAMOND (70 % identity) and the BacMet antibacterial biocide and metal resistance genes database. *Escherichia coli* MAGs were classified by serotype using SerotypeFinder v.2.0 [24]. A phylogenomic tree was generated from the high-quality dereplicated MAGs using PhyloPhlAn v.3.0 by aligning 400 universal marker genes [25]. The relative abundance of each dereplicated MAG in each sample was determined using CoverM v.0.6.1 (https://github.com/wwood/CoverM).

### Statistical analysis

Differentially abundant microbial species, ARGs, copper and zinc resistance genes, CAZymes, and MAGs between the conventionally and pasture-raised pigs were identified with MaAsLin 2 v.1.10.0 [26] in R 4.2.0. Only those microbial species with an overall percentage relative abundance of at least 0.1 % within pigs of the same production phase were included in this analysis. For ARGs, copper and zinc resistance genes, and CAZymes, only those features present in at least 25 % of the samples were included. Permutational multivariate analysis of variance (PERMANOVA) of the Bray–Curtis dissimilarities was calculated in vegan 2.6-2 to determine the effect of farm type on the structure of the microbial community and resistome. The correlations between the microbiome (species) and the resistome (ARGs) and the microbiome and the CAZyme composition were assessed using Procrustes analysis with the Bray–Curtis dissimilarity non-metric multidimensional scaling (NMDS) ordinations in vegan.

## Results

### The faecal microbiome of conventionally vs. pasture-raised pigs

**Fig. 1.**
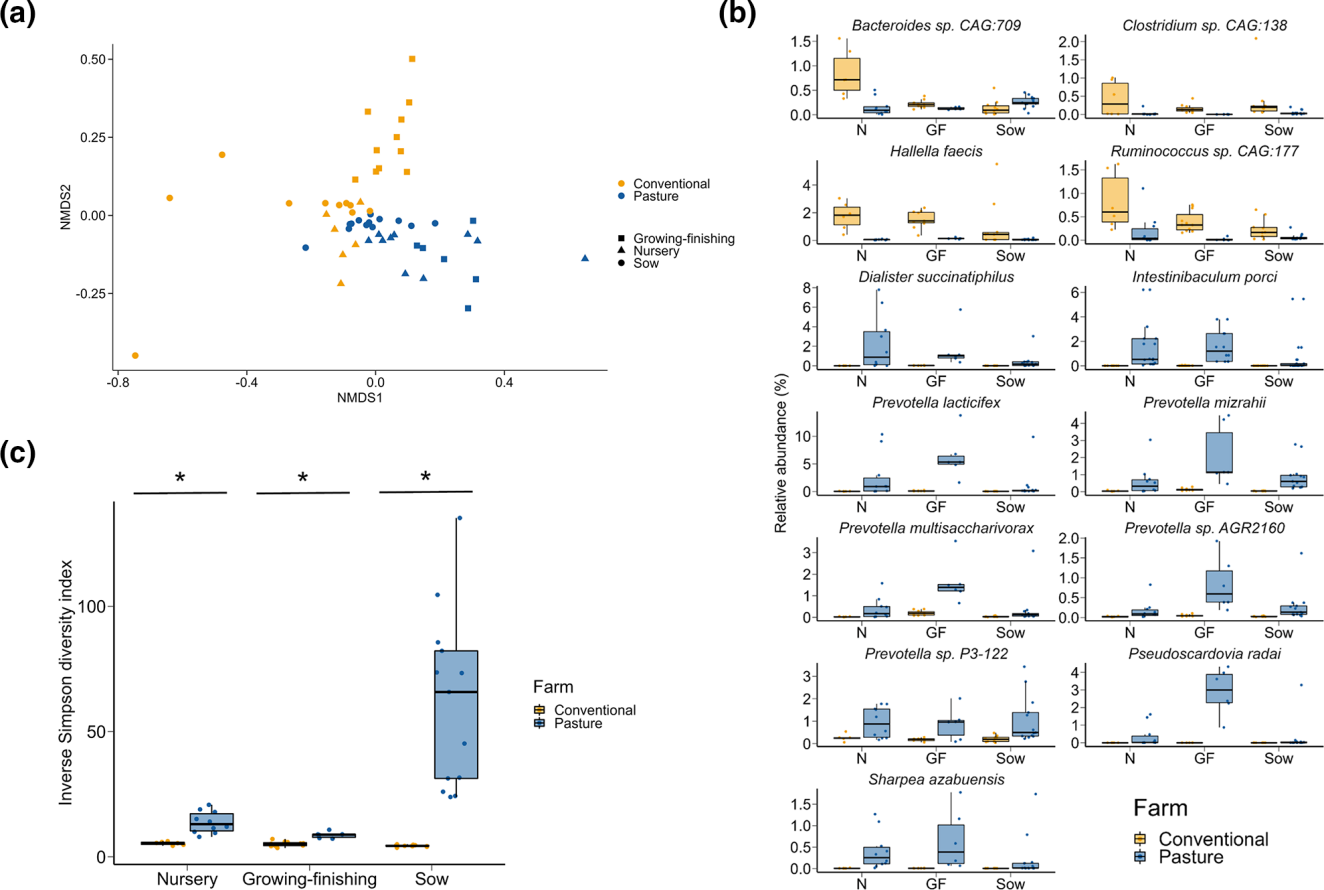
(a) Non-metric multidimensional scaling (NMDS) plot of the Bray–Curtis dissimilarities of microbial species in the faecal microbiomes of conventionally and pasture-raised pigs within three different production phases. (**b**) Percentage relative abundance of bacterial species that were differentially abundant in the faecal microbiomes of conventionally vs. pasture-raised pigs within all three production phases (false discovery rate <0.05). N=nursery; GF=growing-finishing. (**c**) Inverse Simpson diversity index values for microbial species in the faecal microbiomes of conventionally and pasture-raised pigs within three different production phases. *Significantly different means (*P*<0.05).

The faecal microbiome of the two groups of pigs differed significantly at the species level (Fig. 1a; PERMANOVA: *R* ^2^=0.27, *P*<0.001). Among the 141 bacterial species with an overall relative abundance of at least 0.1 % in one of the three production phases, 111 were differentially abundant between the conventionally and pasture-raised pigs (Table S1, available in the online version of this article). *Dialister succinatiphilus *, *Intestinibaculum porci, Prevotella mizrahii, Pseudoscardovia radai* and *Sharpea azabuensis* were among the nine bacterial species that were consistently enriched in pasture pigs and *Bacteroides* sp. CAG-709, *Clostridium* sp. CAG-138, *Hallella faecis* and *Ruminococcus* sp. CAG-177 were relatively more abundant in all pigs raised conventionally (*P*<0.05; Fig. 1b). Although *Prevotella copri* was enriched in the conventional growing-finishing pigs, it was the bacterial species with the highest relative abundance overall in both groups of pigs. Other relatively abundant (>0.3 %) bacterial species in the gut microbiomes of both conventional and pasture pigs were *Oscillibacter valericigenes* and *Prevotella stercorea* (data not shown). Notably, pasture-raised pigs had significantly greater gut microbial species diversity (Fig. 1c).

**Fig. 2.**
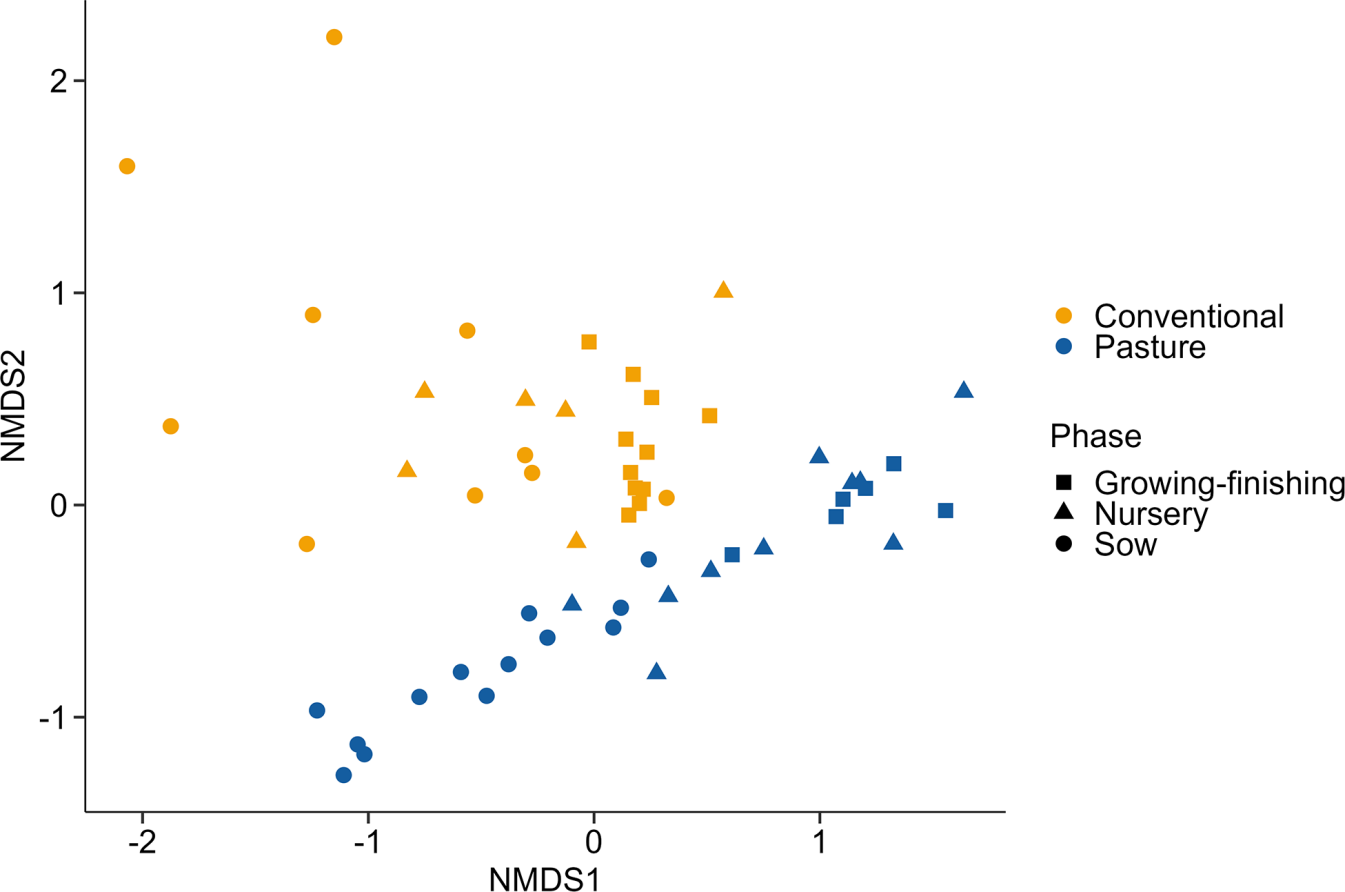
Non-metric multidimensional scaling (NMDS) plot of the Bray–Curtis dissimilarities of carbohydrate-active enzyme (CAZymes) genes in the faecal microbiomes of conventionally and pasture-raised pigs within three different production phases.

CAZymes are enzymes involved in the degradation and synthesis of carbohydrates and are grouped into six different families based on their amino acid sequence similarity: auxiliary activities (AAs), carbohydrate esterases (CEs), carbohydrate-binding modules (CBMs), glycoside hydrolases (GHs), glycosyltransferases (GTs) and polysaccharide lyases (PLs) [27]. Here, the CAZyme profiles of the conventionally and pasture-raised pigs also differed significantly from each other (Fig. 2; PERMANOVA: *R* ^2^=0.13, *P*<0.001). Of the 209 CAZyme families analysed, 111 were differentially abundant between the two groups of pigs within one of the production phases. And of those 111 CAZyme families, 14 were differentially abundant within all three production phase groups (Table S2). Within the microbiomes of conventional pigs, there was a consistent enrichment of GH20 (galactosidases), GH31 (glucosidases) and GH68 (levansucrases) families while GH5_2 (endoglucanases), GT111 (galactofuranosyltransferases) and PL1 (pectinases) were relatively more abundant in all pasture-raised pigs (*P*<0.05). Other CAZyme families of note that were enriched in the conventionally raised growing-finishing pigs and sows included GH29 (fucosidases) and GH33 (sialidases), which are associated with mucin degradation. Not surprisingly, the microbiome (species level) was significantly correlated with the CAZyme profiles (Procrustes: *R* ^2^=0.62; *P*=0.0001)

### The resistome of conventionally vs. pasture-raised pigs

**Fig. 3.**
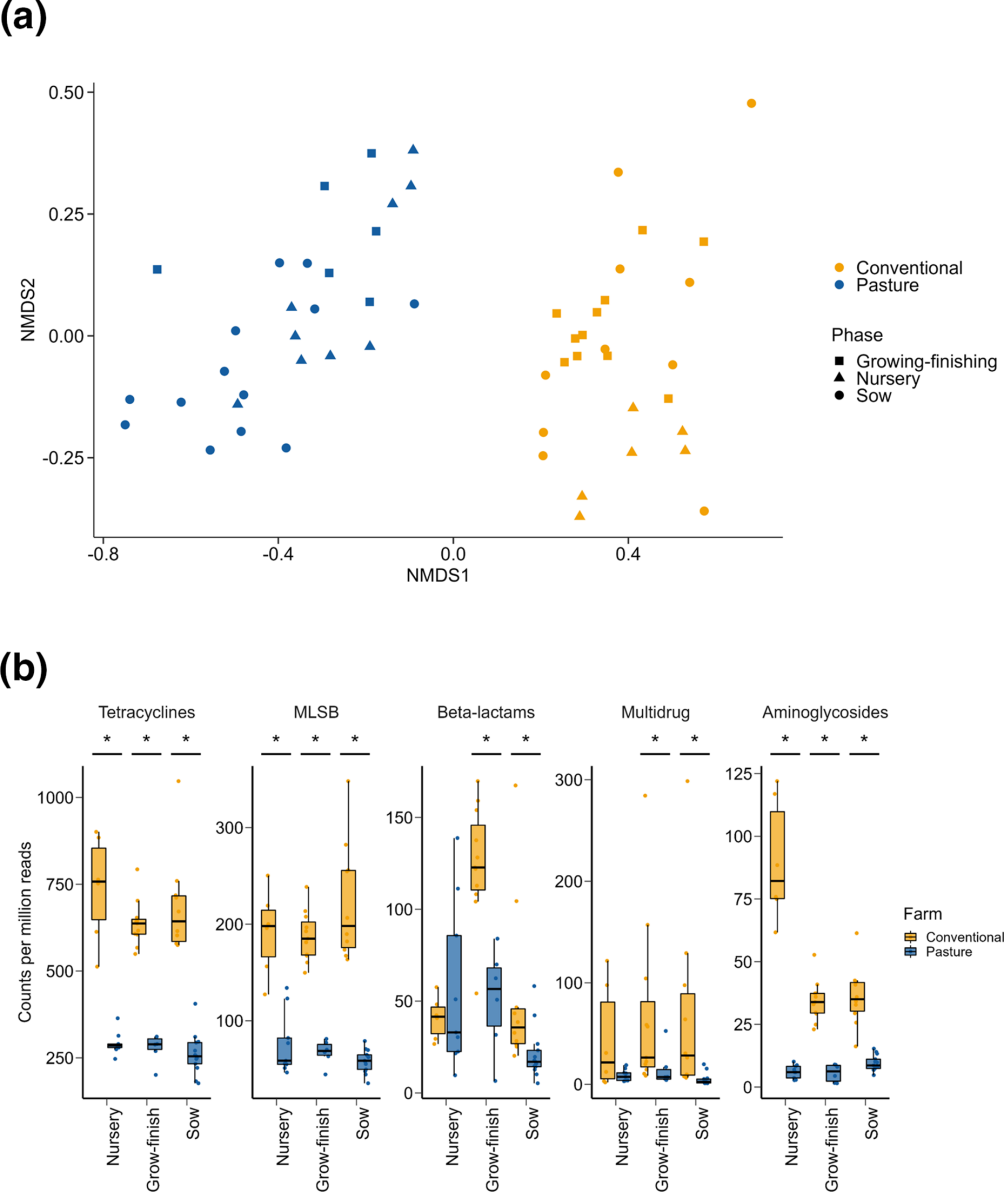
(a) Non-metric multidimensional scaling (NMDS) plot of the Bray–Curtis dissimilarities of antimicrobial resistance genes in the faecal microbiomes of conventionally and pasture-raised pigs within three different production phases. (**b**) Copies per million reads for antimicrobial resistance genes grouped into the antimicrobial classes that they confer resistance to. *Significantly different means (*P*<0.05).

**Fig. 4.**
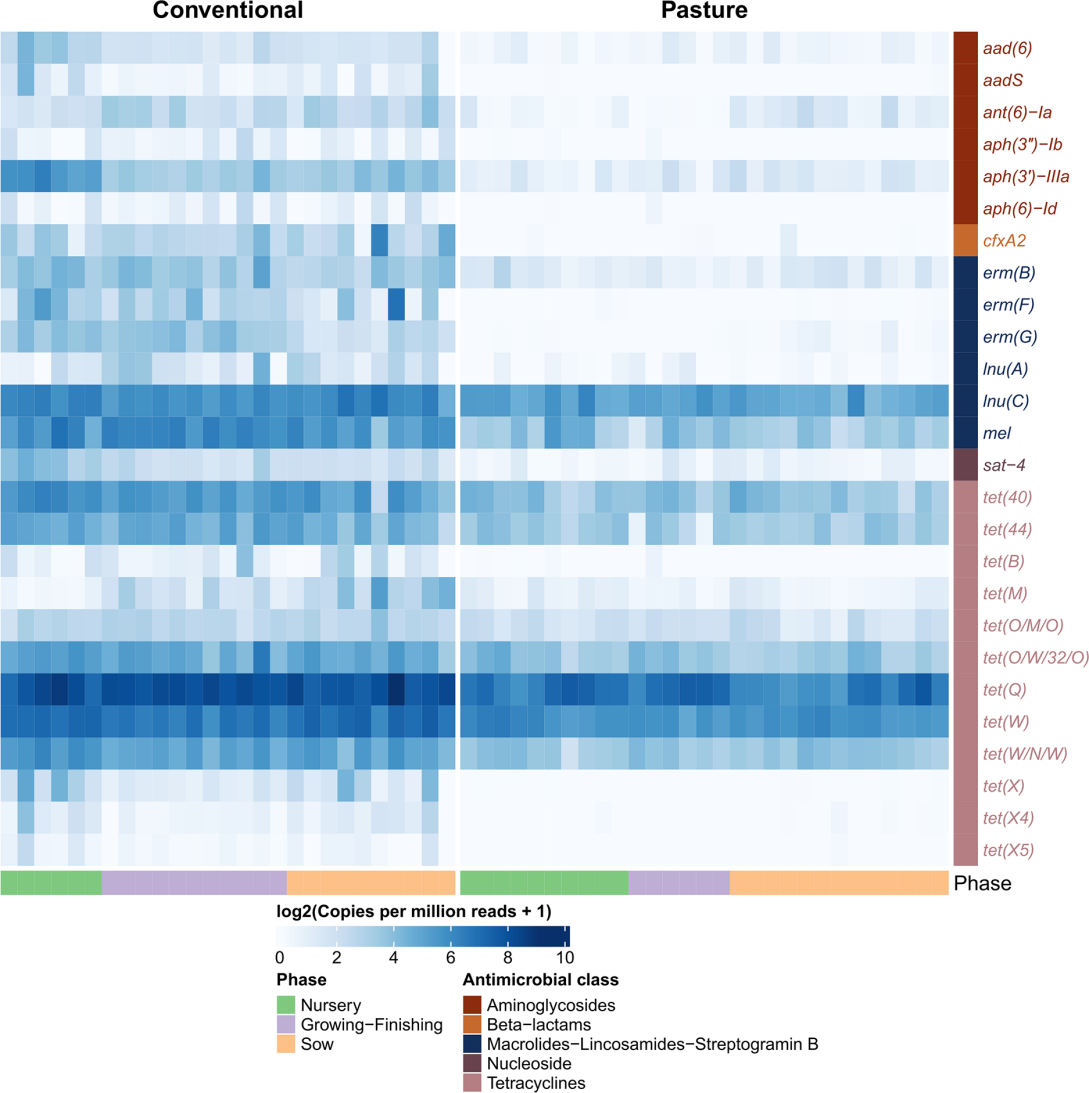
Heat map of the log_2_-transformed copies per million reads for antimicrobial resistance genes that were differentially abundant in the faecal metagenomes of conventionally vs. pasture-raised pigs within three different production phases (false discovery rate <0.05).

The conventionally and pasture-raised pigs also had very different resistome profiles based on the relative abundance of ARGs (Fig. 3a; PERMANOVA: *R* ^2^=0.48, *P*<0.001). In addition, the relative abundance of nearly all classes of ARGs was significantly higher in the conventional group compared with the pasture pigs (Fig. 3b; Table S3; P *P*<0.05). There were 271 individual ARGs identified in at least 25 % of samples within one of the three production phases and 155 of these ARGs were differentially abundant between the two groups of pigs (Table S4). In addition, of these 155 ARGs, 131 were relatively more abundant in the pigs raised under conventional conditions (*P*<0.05). A number of the ARGs consistently enriched in the conventionally raised pigs were those that are frequently abundant in the pig gut such as *aph(3')-IIIa*, *erm*(B), *tet*(Q) and *tet*(W) (Fig. 4). There was also a significant correlation between the faecal microbiome and resistome (Procrustes: *R* ^2^=0.55; *P*=0.0001).

Copper and zinc are typically included in pig diets at concentrations that exceed dietary requirements to control bacterial infections and improve growth [28]. However, the inclusion of dietary copper and zinc at these levels may co-select for ARGs located on the same mobile genetic element [29]. In the present study, the conventionally raised pigs received feed supplemented with supranutritional concentrations of copper (135 mg kg^–1^ feed) and zinc (455 mg kg^–1^) during the nursery phase. Therefore, we also screened the metagenomic sequences for genes associated with copper and zinc resistance. Although there were no copper or zinc resistance genes that were differentially abundant in the microbiomes of growing-finishing pigs, the copper resistance gene *tcrB* was relatively more abundant in the nursery conventional pigs and 28 copper (*cusABCFRS; tcrAB*) and zinc resistance genes (e.g. *zitB*) were enriched in the conventionally raised sows (Table S5).

### Metagenome-assembled genomes

**Fig. 5.**
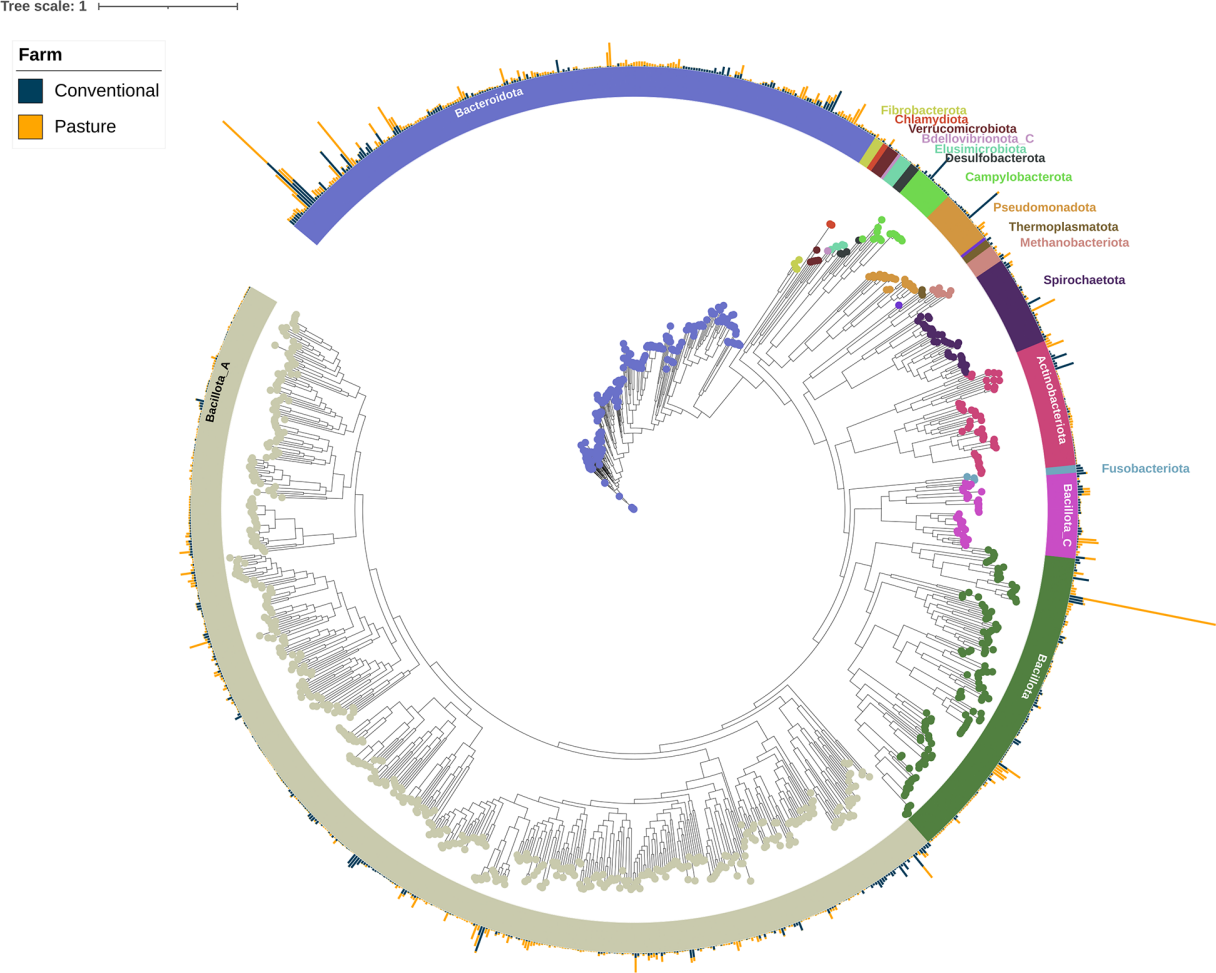
Maximum-likelihood phylogenetic tree of the metagenome-assembled genomes (MAGs) based on the alignment of 400 marker genes. MAGs are coloured and labelled by GTBD-Tk assigned phyla. The outer bars display the percentage relative abundance (0 –3.7 %) of each MAG in the conventionally and pasture-raised pig faecal samples.

Metagenomes from each group of pigs were assembled and binned into MAGs which were then dereplicated at 99 % average nucleotide identity, resulting in 1043 MAGs with ≥90 % completeness and ≤5 % contamination (Fig. 5). These non-redundant MAGs represented 357 genera and 446 species (Table S6). There were 37 and 356 MAGs that were not assigned to a genus or species, respectively, and thus may represent potentially novel genera and species. Furthermore, only 124 MAGs (11.9 %) were assigned to an archaeal or bacterial species with a cultured representative. The most frequently identified bacterial species within the 1043 MAGs were *Sodaliphilus* sp004557565 (12 MAGs) and *Collinsella* sp002391315 (11 MAGs). Four *E. coli* MAGs were recovered and further classified using *in silico* serotyping into three serotypes: H12, O98:H10 and O15:H45.

Reads from each metagenomic sample were also mapped back to the MAGs with 56.4±0.9 % (sem) of the reads aligning to one of the MAGs (Table S7). There were 35 MAGs that were exclusive to the conventionally raised pigs including those classified as *Campylobacter coli *, *Campylobacter hominis* and *Porphyromonas somerae *. Two *Lactobacillus porci* MAGs were among the 17 MAGs recovered only from the pasture-raised pig faecal metagenomes. There were also two *Pseudoscardovia* spp. MAGs identified in 93.1 % of the faecal samples from pasture-raised pigs that were only detected in one conventionally raised sow. Twelve MAGs were differently abundant between the conventionally and pasture-raised pigs within all three production phases (Fig. S1). Similar to the unassembled short reads, these MAGs included those classified as *I. porci* and *Sharpea azabuensis* which were relatively more abundant in the pasture pigs and *Limosilactobacillus reuteri *, *Prevotella* sp002300055 and *Cryptobacteroides* sp000433355 MAGs were enriched in all phases of the conventional pigs. In terms of similarities, three *Prevotella copri* MAGs were among the 10 MAGs with the highest relative abundance in both groups of pigs.

Assembling and binning ARGs is difficult as they are frequently located on mobile genetic elements like plasmids which often have different DNA sequence properties than the host genome [30]. Therefore, as expected, far fewer ARGs were detected in the MAGs compared with the unassembled short reads. However, several *tet* genes were identified within the MAGs (Table S8). The most widely distributed *tet* genes were *tet*(Q) (15 MAGs), *tet*(36) (11 MAGs) and *tet*(W/N/W) (8 MAGs). The rRNA methylase genes *erm*(B) and *erm*(X), conferring resistance to macrolides, were detected in a *Streptococcus suis* MAG and a *Corynebacterium* sp. MAG, respectively. Other ARGs of interest included *aph(3')-IIIa* (aminoglycosides) in a *Campylobacter lanienae* MAG, *bla*_OXA-193_ (beta-lactams) in a *Campylobacter coli* MAG and *cfxA2* (beta-lactams) in two *Sodaliphilus* sp004557565 MAGs. Two *Aerococcus* sp. MAGs that were relatively more abundant in the conventional sow microbiomes were found to be carrying the repressor gene, *tcrY*, from the copper resistance operon *tcrYAZB*, and one of these MAGs also had the *tcrB* gene, which encodes a copper resistance efflux ATPase.

SCFAs are produced from non-digestible carbohydrates by certain members of the microbiome in the lower gastrointestinal tract of monogastric animals such as pigs. These SCFAs, which include acetate, butyrate and propionate, provide energy for the host and have anti-inflammatory properties as well. Here, genes for butyrate production via the butyryl-CoA:acetate CoA-transferase or butyrate kinase pathways were encoded by 84 and 114 MAGs, respectively (Table S9). *Megasphaera elsdenii* and *Sodaliphilus pleomorphus* MAGs carrying genes for butyrate production were also among those significantly associated with conventionally raised growing-finishing pigs. In addition, several *Fusobacterium* and *Porphyromonas* spp. MAGs enriched in the conventional farm sows encoded genes for one of the two butyrate pathways. Two *Alloprevotella* sp004557185 MAGs with the butyrate kinase genes were relatively more abundant in the pasture-raised nursery phase pigs. Genes for acetate production were identified in the large majority of MAGs (84.7 %) while those coding for propionate were found in only 69 MAGs.

CAZymes involved in the degradation of amorphous cellulose, arabinose and mixed-linkage glucans were encoded by at least 50 % of the MAGs (Table S9). The greatest number of CAZymes and CAZyme families were found in MAGs classified as *Alistipes senegalensis *, *Parabacteroides faecavium*, *Phocaeicola plebeius *, *Phocaeicola vulgatus* and *Prevotella* sp004554665 (Table S10). There were also 432 CAZyme families detected among all the MAGs, including 164 GH families of which GH2, GH3, GH13, GH23 and GH25 were most prevalent.

## Discussion

The faecal microbiomes and resistomes of pigs raised on a conventional farm were significantly different from those of pigs raised outdoors on pasture. This is not unexpected given the large differences between the two production systems and demonstrates the large impact that management practices can potentially have on the pig gut microbiome and resistome. Pigs on the conventional farm were housed completely indoors and in pens with other pigs as per industry standards whereas the pasture-raised pigs were reared entirely outdoors at a much lower stocking density. There were also differences in the breeds of pigs on the two farms as the pasture-raised farm used pig breeds better suited to living outdoors in Canada year-round. Although both groups of pigs were fed a grain-based diet, pigs on pasture will often use their snouts to root in the soil and so it is likely that they are ingesting plants, soil and other organic matter.

Using both short, unassembled metagenomic reads and MAGs, we identified a number of bacterial species strongly associated with one of the farm types. Some of the bacterial species with a higher relative abundance in the conventionally raised pigs were those that can be potentially pathogenic in swine such *Streptococcus suis* and *Campylobacter coli *, although these species are often recovered from healthy pigs as well [31]. Other bacterial species enriched in the conventionally raised pigs included *L. reuteri *, *Prevotella* sp002300055, and *Treponema succinifaciens *. Supplementation of swine feed with *L. reuteri* has been linked with improved growth performance and a reduction in certain bacterial pathogens in the pig gut [32–34], and *T. succinifaciens* has also been reported to be associated with the gut microbiome of pigs with higher feed efficiency [35]. *Prevotella* sp002300055 is a placeholder species that we previously identified as abundant in swine gut metagenomes from multiple countries [36].

The gut microbiomes of pigs raised on pasture were enriched with several bacterial species that are not typically abundant in conventionally raised swine. These included two *Pseudoscardovia* spp. MAGs that were not identified in any of the conventional nursery or growing-finishing pigs and in only one conventional sow. Presently, there are two recognized *Pseudoscardovia* species, *Pseudoscardovia radai* and *Pseudoscardovia suis *, both of which were originally characterized using isolates from the gastrointestinal tract of wild boars [37, 38]. Therefore, *Pseudoscardovia* spp., which are closely related to the bifidobacteria, appear to be strongly associated with pigs in the outdoor environment. Two related lactic acid-producing bacterial species, *I. porci* and *Streptococcus azabuensis*, were also significantly enriched in the faecal microbiomes of pasture-raised pigs. *Streptococcus azabuensis* has been reported to be relatively more abundant in young healthy pigs vs. those with diarrhoea [39] and has also been linked with reduced methane emissions in ruminants [40]. *I. porci* is a relatively newly identified bacterial species that was originally isolated from the small intestine of a pig [41] and similar to *S. azabuensis *, it has been associated with lower methane emissions in cattle [42].

Although *Prevotella copri* was enriched in the conventional sow gut microbiome, it was the bacterial species with the highest relative abundance in both conventionally and pasture-raised pigs and therefore appears to be well-adapted to the domestic pig gut regardless of diet and environment. In pigs, *Prevotella copri* has been associated with the post-weaning phase [4, 43] and fat accumulation [44]. However, *Prevotella copri* is a genetically diverse species that has recently been proposed to contain at least four distinct clades [45] and therefore the *Prevotella copri* strains in the conventionally and pasture-raised pigs are especially unlikely to be shared given their dissimilar gut microbiomes.

Functionally, the conventional and pasture pig gut microbiomes were also significantly different based on their CAZyme profiles. CAZymes synthesize and degrade carbohydrates and are prevalent in the gut microbiomes of mammals [46]. In gut bacteria, the GHs are the major CAZyme family involved in the breakdown of dietary glycans [47] and here several GH families were differentially abundant between the two pig farms. Notably, certain CAZyme families associated with host mucin degradation such as GH20 (galactosidases), GH29 (fucosidases) and GH33 (sialidases) were enriched in the microbiomes of the conventionally raised pigs. Bacteria that produce these CAZymes can release oligo- and monosaccharides from mucin glycans found in the gut mucosa which can then be used by other gut bacteria to produce metabolites such as SCFAs through a process called cross-feeding [48].

There were also 19 MAGs enriched in one of the three conventionally raised pig production groups that carried genes coding for CAZymes within the GH20, GH29 and GH33 families. Included among these MAGs were two *Sodaliphilus pleomorphus* MAGs and several others from uncultured species such as *Alloprevotella* sp004552155, *Cryptobacteroides* sp000433355 and *Prevotella* sp000434975. *Sodaliphilus pleomorphus* is a relatively newly identified bacterial species within the *Muribaculaceae *, a family associated with mucin degradation [49]. It is possible that the less diverse diet of conventional pigs, compared to that of pigs raised outdoors, favours bacteria that can metabolize host mucins for energy.

Conventionally raised pigs almost always have a large background of certain antimicrobial-resistant bacteria and ARGs even in the absence of direct antimicrobial exposure [2, 50, 51]. Here, compared to the pasture-raised pigs, the abundance of ARGs within nearly all antimicrobial classes was significantly higher in the conventionally raised pigs. These ARGs included many that are typically abundant in commercial pigs such as *aph(3')-IIIa*, *cfxA*2, *erm*(B), *erm*(F), *lnu*(C), *tet*(44), *tet*(Q), *tet*(W) and *tet*(X) [2, 52–54]. Some of these ARGs were also assembled and binned into specific MAGs, thus providing taxonomic information. For example, *erm*(B) was linked to a *S. suis* MAG and *tet*(44) was identified in a *Terrisporobacter* sp. MAG, both of which were relatively more abundant in conventionally raised swine. As mentioned earlier, *Streptococcus suis* is a potential pathogen in pigs that can cause arthritis, endocarditis and meningitis, although it is frequently found in healthy pigs as well [55]. A *Campylobacter coli* MAG belonging to sequence type ST900 and enriched in the conventionally raised sows was also carrying *bla*_OXA-193_, a beta-lactamase gene. *Campylobacter coli* is prevalent in healthy pigs but has also been implicated in foodborne disease in humans, although less commonly than *Campylobacter jejuni* [56]. The *bla*_OXA-193_ gene has been reported to be relatively common in *Campylobacter coli* isolates from multiple sources, including pigs [57].

Although the conventional pigs were not administered any antimicrobials during the study, similar to most conventional swine operations in North America, antimicrobials were used in the facility in the past to promote growth and are still used on a short-term metaphylaxis basis to prevent the spread of disease. Not surprisingly, the relative abundance was also highest for those ARGs that confer resistance to antimicrobials with the longest history and prevalence of use in swine production in North America such as the beta-lactams (e.g. ceftiofur, penicillin), macrolides-lincosamides-streptogramin B (MLS_B_) (e.g. lincomycin, tulathromycin, tylosin) and tetracyclines (e.g. chlortetracycline, oxytetracycline) [58, 59]. These ARGs appear to be well integrated into the pig gut microbiome without imposing a serious fitness cost to the bacteria that harbour them. Therefore, reducing the abundance of these ARGs probably requires significantly altering the gut microbiome itself as there was a strong association between the species-level composition and the resistome here. Conventionally raised pigs may also be exposed to biocides and disinfectants in their environment as well as high levels of dietary copper and zinc, all of which can co-select for ARGs [60]. Indeed, sows on the conventional farm had a significantly higher relative abundance of a number of different copper and zinc resistance genes, implying there may be some association with the use of these metals at supra-nutritional levels and certain ARGs.

## Conclusion

The gut microbiomes of conventionally and pasture-raised pigs differed significantly from each other with certain bacterial species strongly associated with each group. This included bacterial species such as *Pseudoscardovia* spp. which are rarely detected in conventionally raised swine but were prevalent in the pasture-raised pigs. Importantly, a large number of ARGs were significantly enriched in the gut microbiome of pigs on the conventional farm. Although reducing antimicrobial resistance in commercial food-producing animals such as pigs is very challenging, this study suggests that management practices may potentially make a large difference in this regard.

## Supplementary Data

Supplementary material 1

Supplementary material 2
