# Supplementary material for: The gut microbiome and resistome of conventionally vs. pasture-raised pigs
Source: Microb Genom. 2023 Jul 13;9(7):mgen001061. doi: 10.1099/mgen.0.001061 (PMC10438820; doi:10.1099/mgen.0.001061)
Supplement: Supplementary material 1 [file mgen-9-1061-s001.pdf]

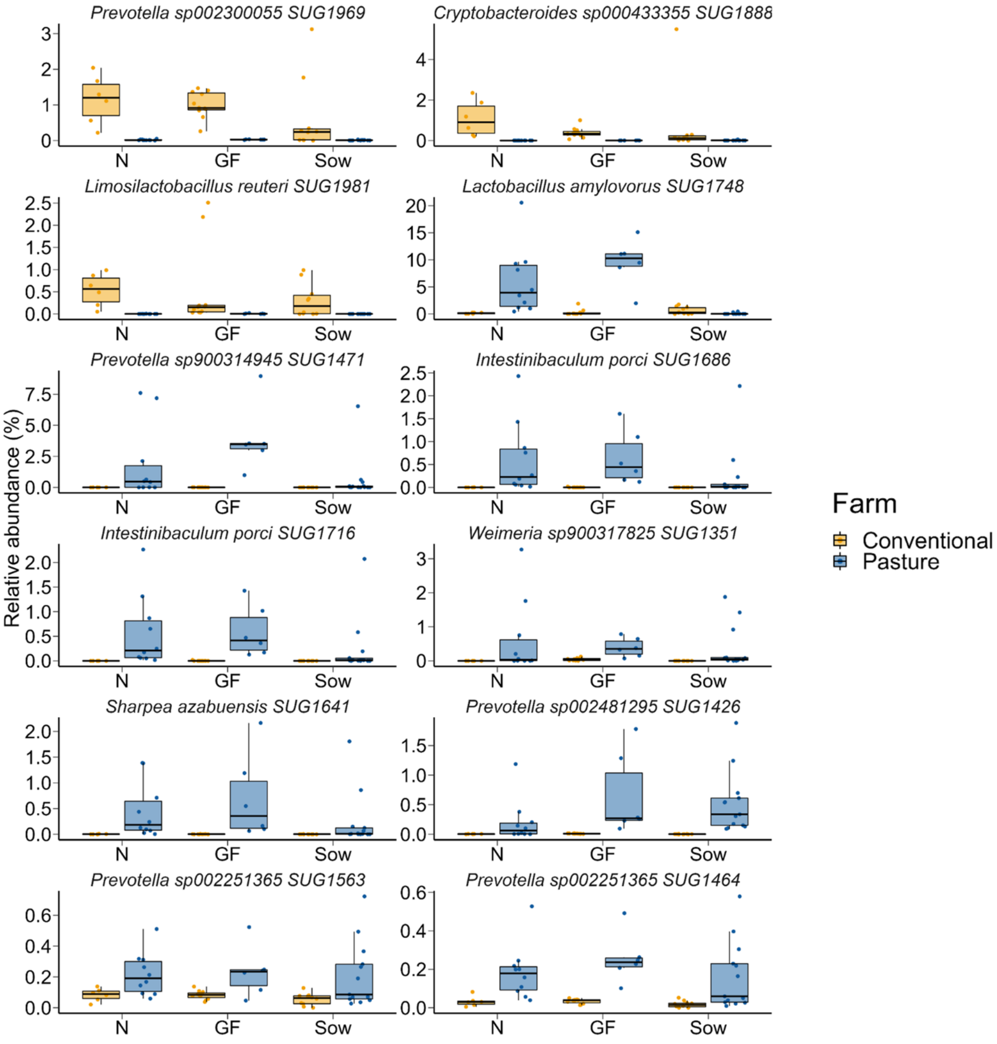

Figure S1. Percent relative abundance of metagenome-assembled genomes that were differentially abundant in the gut microbiomes of conventionally- and pasture-raised pigs within all three production phases. N = nursery; GF = growing-finishing.
